# Contributions of Artificial Intelligence Reported in Obstetrics and Gynecology Journals: Systematic Review

**DOI:** 10.2196/35465

**Published:** 2022-04-20

**Authors:** Ferdinand Dhombres, Jules Bonnard, Kévin Bailly, Paul Maurice, Aris T Papageorghiou, Jean-Marie Jouannic

**Affiliations:** 1 Fetal Medicine Department Armand Trousseau University Hospital Sorbonne University Paris France; 2 Laboratory in Medical Informatics and Knowledge Engineering in e-Health Institut National de la Santé et de la Recherche Médicale Sorbonne University Paris France; 3 Institute for Intelligent Systems and Robotics Sorbonne University Paris France; 4 Oxford Maternal & Perinatal Health Institute Green Templeton College Oxford United Kingdom

**Keywords:** artificial intelligence, systematic review, knowledge bases, machine learning, obstetrics, gynaecology, perinatology, medical informatics

## Abstract

**Background:**

The applications of artificial intelligence (AI) processes have grown significantly in all medical disciplines during the last decades. Two main types of AI have been applied in medicine: symbolic AI (eg, knowledge base and ontologies) and nonsymbolic AI (eg, machine learning and artificial neural networks). Consequently, AI has also been applied across most obstetrics and gynecology (OB/GYN) domains, including general obstetrics, gynecology surgery, fetal ultrasound, and assisted reproductive medicine, among others.

**Objective:**

The aim of this study was to provide a systematic review to establish the actual contributions of AI reported in OB/GYN discipline journals.

**Methods:**

The PubMed database was searched for citations indexed with “artificial intelligence” and at least one of the following medical subject heading (MeSH) terms between January 1, 2000, and April 30, 2020: “obstetrics”; “gynecology”; “reproductive techniques, assisted”; or “pregnancy.” All publications in OB/GYN core disciplines journals were considered. The selection of journals was based on disciplines defined in Web of Science. The publications were excluded if no AI process was used in the study. Review, editorial, and commentary articles were also excluded. The study analysis comprised (1) classification of publications into OB/GYN domains, (2) description of AI methods, (3) description of AI algorithms, (4) description of data sets, (5) description of AI contributions, and (6) description of the validation of the AI process.

**Results:**

The PubMed search retrieved 579 citations and 66 publications met the selection criteria. All OB/GYN subdomains were covered: obstetrics (41%, 27/66), gynecology (3%, 2/66), assisted reproductive medicine (33%, 22/66), early pregnancy (2%, 1/66), and fetal medicine (21%, 14/66). Both machine learning methods (39/66) and knowledge base methods (25/66) were represented. Machine learning used imaging, numerical, and clinical data sets. Knowledge base methods used mostly omics data sets. The actual contributions of AI were method/algorithm development (53%, 35/66), hypothesis generation (42%, 28/66), or software development (3%, 2/66). Validation was performed on one data set (86%, 57/66) and no external validation was reported. We observed a general rising trend in publications related to AI in OB/GYN over the last two decades. Most of these publications (82%, 54/66) remain out of the scope of the usual OB/GYN journals.

**Conclusions:**

In OB/GYN discipline journals, mostly preliminary work (eg, proof-of-concept algorithm or method) in AI applied to this discipline is reported and clinical validation remains an unmet prerequisite. Improvement driven by new AI research guidelines is expected. However, these guidelines are covering only a part of AI approaches (nonsymbolic) reported in this review; hence, updates need to be considered.

## Introduction

The foundations of artificial intelligence (AI) as a discipline were established in the 1950s, under the hypothesis formulated by John McCarthy as “Every aspect of learning or any other feature of intelligence can in principle be so precisely described that a machine can be made to simulate it” [1]. Developing AI was a 3-fold challenge: collect an unprecedented amount of data for training and validation of algorithms, build computers with sufficient computational power, and create algorithms to simulate human intelligence functions (eg, reasoning, learning, adaptation, vision, interaction).

At the dawn of the 21st century, all 3 challenges have been taken up in many fields, leveraging different types of AI approaches. Two general directions in AI research approaches have been pursued: symbolic approaches and nonsymbolic approaches. The symbolic AI approach, also known as “Good Old-Fashioned AI” (GOFAI) [2], encompasses formal logic, knowledge representation, and rule-based and semantic reasoning. These approaches are generally explainable and human-readable, need human curation and design, and do not rely on a large amount of data to develop. The first GOFAI-related publications in the field of medicine emerged 60 years ago [3], and these approaches provided the first significant results with expert systems [4,5] and are now widely used in knowledge-based systems [6,7], mostly through the application of ontologies and semantic web technologies [8-10]. Nonsymbolic AI, defined as intelligence without specific knowledge representations, encompasses various approaches to simulate other human intelligence processes such as learning, perception, and pattern recognition. Machine learning (ML) has become the main approach in this area [11], mostly through algorithms such as artificial neural networks (ANNs), and relies on a large amount of high-quality data to learn, train, and validate, along with significant computational resources. This AI is generally “nonexplainable,” with the process occurring inside ANNs (architecture, layers, and connections) remaining in the form of a “black box” to the users.

**Figure 1:**
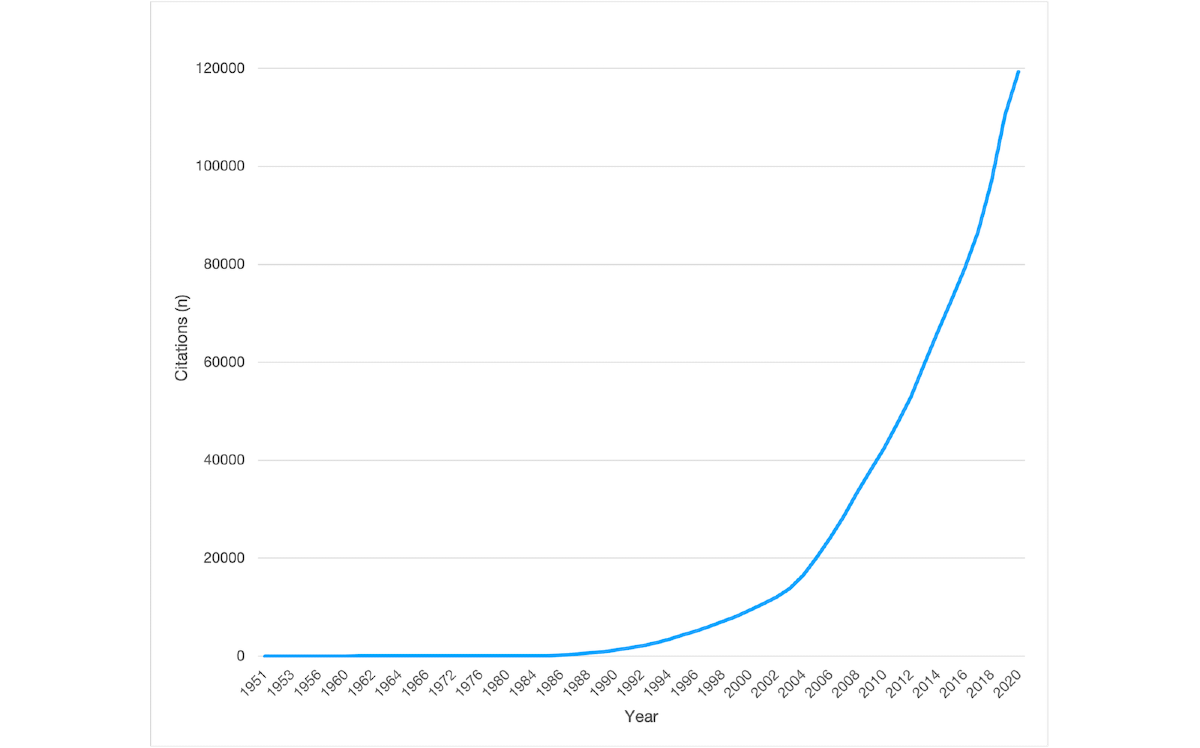
Trend of the 119,325 citations in PubMed indexed with the MeSH (Medical Subject Heading) term “artificial intelligence” between 1951 and 2020.

Publications in AI in medicine have grown rapidly during the last two decades: 119,325 citations are referenced in PubMed, 93% of which have been published since 2000 (Figure 1). The obstetrics and gynecology (OB/GYN) domain represents a wide range of medical activities (obstetrics, fetal medicine, open and endoscopic surgery, ultrasound and other imaging modalities, reproductive biology, and assisted reproductive technologies). These activities are leveraging various types of data (eg, textual data; 2D, 3D, and 4D imaging data; genomic and proteomic data; fetal monitoring data). However, it is only recently that AI concepts (ML principles) were described in an OB/GYN ultrasound imaging journal [12]. Interestingly, the general emergence of AI in the OB/GYN domain, and more specifically in OB/GYN journals, has not been investigated.

Our aim was to establish the actual contributions of AI reported in OB/GYN journals with a systematic review to investigate, within all OB/GYN subdomains, the AI methods, sources of data, and the contribution and validation of the AI processes. Most of the recent publications about AI usually focus on ML, ANNs, and deep learning. In this review, we considered all AI contributions, including both symbolic and nonsymbolic AI.

## Methods

### Design

This systematic review was performed in accordance with the recommended PRISMA (Preferred Reporting Items for Systematic Reviews and Meta-Analyses) guidelines [13]. The PRISMA Checklist for this study is presented in Multimedia Appendix 1.

### Ethics Approval

As no patients were involved in the study, this study was exempted from institutional review board approval.

### Literature Search Strategy

The PubMed database was searched for citations published between January 1, 2000, and April 30, 2020. We used the National Library of Medicine Medical Subject Headings (MeSH) terms to search for citations indexed with “artificial intelligence” and at least one other MeSH term from the OB/GYN domain: “obstetrics”; “gynecology”; “reproductive techniques, assisted”; or “pregnancy.” This search was restricted to English-language publications with an abstract, using the following query: “artificial intelligence” [MeSH Terms] AND (“obstetrics” [MeSH Terms] OR “gynecology” [MeSH Terms] OR “reproductive techniques, assisted” [MeSH Terms] OR “pregnancy” [MeSH Terms]) AND 2000/1/1:2020/4/30 [Date Publication].

The results of the query were considered final on November 30, 2020, to cover all publications with potential delayed indexation in PubMed.

All retrieved citations were classified according to disciplines defined in Web of Science (WoS) for the Journal Citation Reports (JCR) and grouped in the following 9 discipline categories: OB/GYN core disciplines journals, other medical clinical disciplines journals, medical nonclinical disciplines journals, medical genetics/biology disciplines journals, medical imaging journals, medical informatics journals, computer science disciplines journals, engineering disciplines journals, and other science disciplines journals. The detailed disciplines and discipline categories are presented in Table S1 of Multimedia Appendix 2 for all journals.

We included all publications from journals or conference proceedings of the core OB/GYN WoS disciplines, namely *Obstetrics & Gynecology*, *Surgery*, *Oncology*, *Developmental Biology*, *Reproductive Biology*, *Andrology*, or *Urology & Nephrology*. The publications were excluded if no AI process was used in the study. Review, editorial, and commentary articles were also excluded.

The publication selection was independently performed by two researchers (FD, JB) by full-text review to assess the actual use of any AI process in the study. Discrepancies on AI process assessments between reviewers were resolved during meetings with KB and JMJ.

### Data Collection and Analysis

The data collection and the qualitative analysis of the citations comprised six different tasks: (1) classification of publications into 5 OB/GYN domains (obstetrics, gynecology, assisted reproductive medicine, early pregnancy, and fetal medicine), (2) description of the AI method used in the study (eg, ML, knowledge base), (3) description of the AI algorithm used in the study (eg, ANN, support vector machine, bioinformatics knowledge bases), (4) description of the type of data used in the AI process (eg, image data set, omics data set), (5) contribution of the AI process (eg, new algorithm, hypothesis generation, fully functional software), and (6) description of the validation of the AI process (eg, validation on one data set, validation on more than one data set, clinical validation). The statistical synthesis of this systematic review was performed by computing the proportion of publications by groups defined in the qualitative analysis.

The evolution over time of the scientific production related to AI in OB/GYN was assessed by a trend analysis of publications per year during the entire review period for OB/GYN core journals and other science journals. The respective contributions of all scientific disciplines in the retrieved citations were assessed by the analysis of their distribution across all WoS disciplines and the proportion of citations in each of the 9 science discipline categories.

## Results

### Study Selection

**Figure 2:**
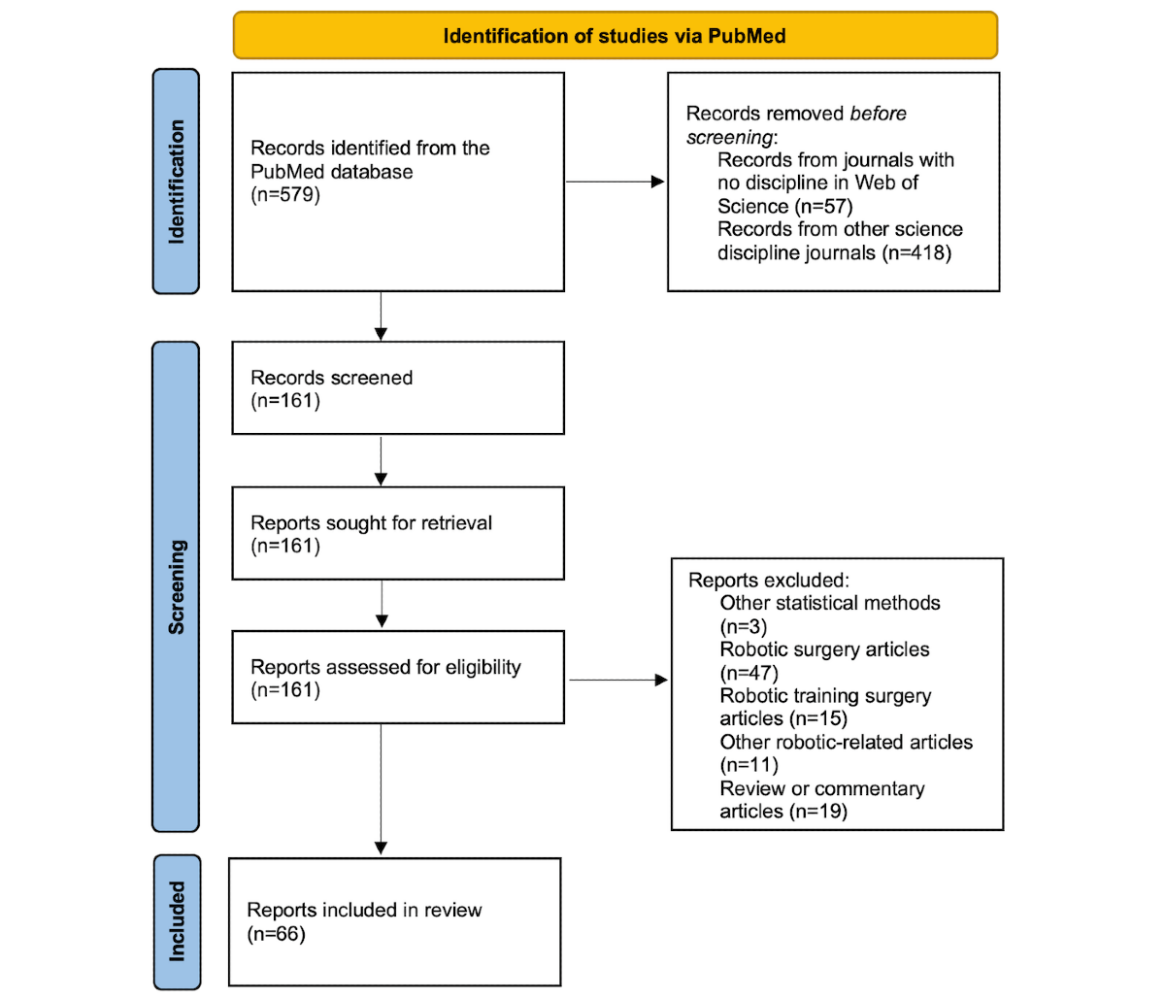
PRISMA (Preferred Reporting Items for Systematic Reviews and Meta-Analyses) 2020 flow diagram for the selection process of the studies included in this review.

The PubMed search retrieved 579 citations. The 161 publications from OB/GYN core disciplines journals were reviewed for eligibility assessment. A total of 66 publications met the selection criteria [14-79]. The flow chart of the publications reviewed is presented in Figure 2.

**Figure 3:**
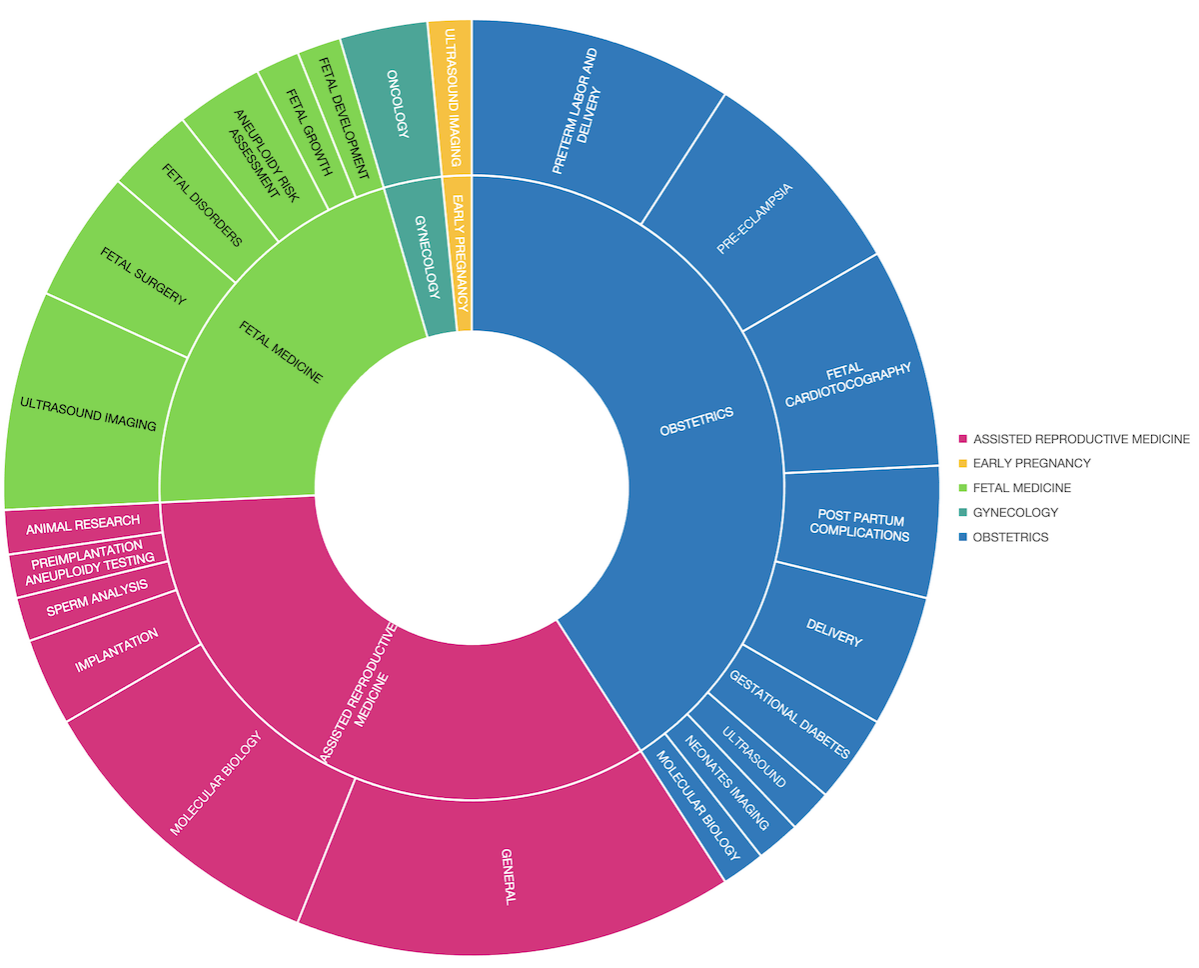
Distribution of the 66 artificial intelligence publications in obstetrics and gynecology journals, across subdomains.

All OB/GYN domains were represented in these selected publications (N=66): obstetrics (n=27, 41%), gynecology (n=2, 3%), assisted reproductive medicine (n=22, 33%), early pregnancy (n=1, 2%), and fetal medicine (n=14, 21%). The detailed distribution of the publications in these domains is presented in Figure 3.

### Description of AI Methods

The two main AI methods in the 66 selected papers were represented by ML methods [14-47,66-70] (59%, n=39) and by knowledge base methods [50-65,71-79] (38%, n=25). In obstetrics and in fetal medicine, ML was more common than other AI methods in 70% (19/27) and 71% (10/14) of the publications, respectively. ML methods and knowledge bases were used in 45% (10/22) and 55% (12/22) of the publications in in the assisted reproductive medicine domain, respectively.

The ML methods comprised mainly ANNs (25/39, 64%) [14-25,27-30,33-35,41,43-45,47,68]. Diverse other ML approaches are used, ranging from classical ML tools such as support vector machine [26] and genetic algorithms [31] to more recent methods such as random forest [38,46] or gradient boosted trees [37,42,67]. Very recently, more evolved and combined neural networks are used in deep learning to process complex data for image segmentation (eg, [41,44]) or classification (eg, [39,43]). The knowledge base methods comprised bioinformatic processes involving mainly Gene Ontology (88%, 22/25) but also other omics knowledge bases, text-mining processes leveraging ontologies, and semantic reasoning processes based on domain ontologies.

The data sets used with all AI methods in the selected studies are detailed in Table 1. ML methods dealt primarily with ultrasound imaging (2D, 3D, video), numerical, and clinical data sets, whereas knowledge base methods dealt mostly with omics data sets.

The contribution of using AI methods were for algorithm development (53%), hypothesis generation (42%), or software development (3%).

When using knowledge base methods, the main AI contribution was to generate hypotheses in physiology or physiopathology (reproduction and implantation, preeclampsia, fetal growth, or breast cancer). When using ML methods, the AI contribution was to build prediction algorithms (implantation success, neonatal outcome, preterm delivery, fetal weight, aneuploidy risk, or postpartum complications). The detailed contributions for all AI methods are presented in Table 2.

Most ML methods were applied to one data set (87%, 34/39) and the use of two or more data sets was less common (13%, 5/39). No external clinical validation of ML methods was identified in the selected articles. Knowledge base methods were applied on one data set in all cases and validated in one clinical study.

**Table 1:** Type of data and artificial intelligence methods used in the 66 selected articles.

Type of data		Articles, n
**Knowledge base method data sets**		
	cDNA^a^/RNA-sequencing	16
	Mixed (clinical and transcriptomic data)	3
	Proteomic/spectrometry	2
	Other: text (publications), imaging (2D ultrasound), mixed (clinical and proteomic data), genomic data repository	4
**Machine learning method data sets**		
	Clinical (numeric/categorical variables)	16
	Numeric (fetal biometry)	4
	Numeric (fetal heart monitoring/FSpO2^b^ data)	4
	Image (microscopy)	3
	Video (fetoscopy)	3
	Image (2D ultrasound)	2
	Other: administrative (numerical/categorical variables), registry (numerical/categorical variables), numeric (electromyography), numeric (maternal EKG^c^), mixed (clinical and genomic data), DNA methylation, proteomic/spectrometry	7
Fuzzy logic data sets: numeric (fetal heart monitoring/FSpO2 data)		1
Other data sets, artificial intelligence method not specified (image dataset: 3D ultrasound)		1

^a^cDNA: complementary DNA.

^b^FSpO2: fetal oxygen saturation.

^c^EKG: electrocardiogram.

**Table 2:** Contributions of artificial intelligence methods used in the 66 selected articles.

Contribution of artificial intelligence methods		Articles, n
**Knowledge base method contributions**		
	Hypothesis generation: ART^a^ techniques/implantation physiology	7
	Hypothesis generation: preeclampsia physiopathology	3
	Hypothesis generation: reproduction physiology	3
	Hypothesis generation: breast cancer physiopathology	2
	Hypothesis generation: fetal growth/development physiology	2
	Method: variant characterization	1
	Method: guided ultrasound image analysis	1
	Other hypothesis generation: pregnancy physiology, diabetes physiopathology, preterm labor physiopathology, recurrent pregnancy loss physiopathology, stem cell profiling, candidate gene/variant	6
**Machine learning method contributions**		
	Algorithm: implantation/ART method success prediction	6
	Algorithm: neonatal outcome prediction	4
	Algorithm: preterm delivery prediction	3
	Algorithm: delivery route prediction	3
	Algorithm: fetal weight/growth abnormalities prediction	3
	Algorithm: aneuploidy prediction/aneuploidy risk assessment	2
	Algorithm: postpartum complications prediction	2
	Other algorithms: gestational age prediction, preeclampsia prediction, blastocyst grading, classification of lung disorders, muscle image segmentation	5
	Method: fetoscopic images annotation	2
	Other methods: placental blood vessels detection, preterm outcome risk assessment, fertility phenotyping	3
	Hypothesis generation: diabetes physiopathology, fetal alcohol disorder spectrum physiopathology, gastroschisis physiopathology, coagulation physiopathology, uterus physiology	5
	Prototype software: ART success prediction	1
Fuzzy logic method contributions: functional software (3D fetal heart analysis)		1
Other contributions, artificial intelligence method not specified: algorithm (neonatal outcome prediction)		1

^a^ART: assisted reproductive technology.

### General Trend in AI Publications

**Figure 4:**
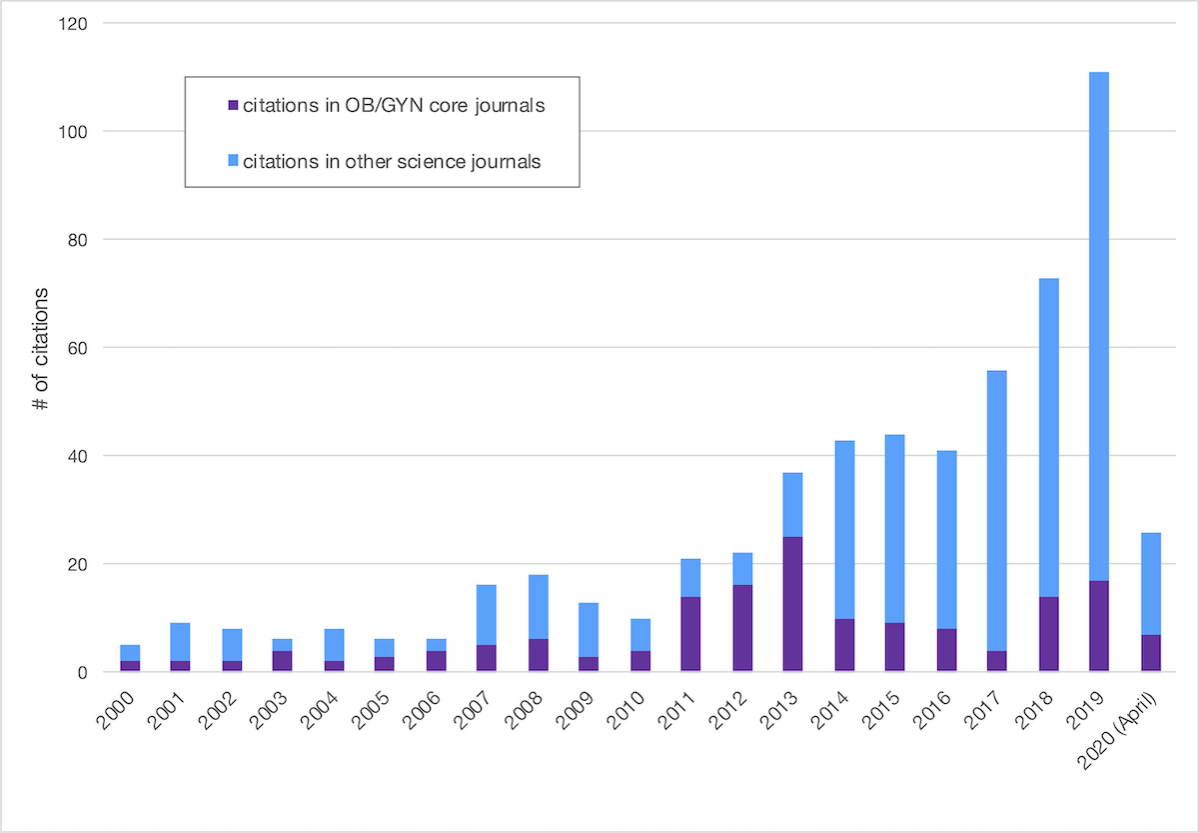
Trends in PubMed artificial intelligence citations between 2000 and 2020 in obstetrics and gynecology (OB/GYN) journals and in other scientific disciplines journals.

We observed a significant rising trend in the scientific production over the last two decades, mainly outside the OB/GYN core journals (Figure 4). A total of 67 science disciplines covered this scientific production (579 PubMed indexed citations), 18% of which were in OB/GYN core disciplines journals. The distribution of citations in the other discipline categories is shown in Table 3. The science discipline was not defined in WoS/JCR for 6% of the citations.

**Table 3:** Distribution of the 579 PubMed artificial intelligence citations between 2000 and 2020 among the 67 science disciplines.

Science disciplines	Articles (N=874)^a^, n (%)
OB/GYN^b^ core disciplines	161 (18.4)
Medical imaging discipline	50 (5.7)
Other medical clinical discipline	47 (5.4)
Medical nonclinical discipline	115 (13.2)
Medical informatics discipline	60 (6.9)
Medical genetics/biology disciplines	58 (6.6)
Engineering disciplines	79 (9.0)
Computer science disciplines	66 (7.6)
Other science disciplines	181 (2.1)
Absence of discipline in Web of Science	57 (6.5)

^a^Since some citations are multidisciplinary, the total is higher.

^b^OB/GYN: obstetrics and gynecology.

## Discussion

### Main Findings

In this review, we have demonstrated that AI contributions are emerging in OB/GYN journals and that a wide range of AI approaches (symbolic and nonsymbolic) are applied across all OB/GYN subdomains. ML is the most common nonsymbolic AI approach (59%) and articles are based mainly on ANNs (64%). Knowledge bases are the most common symbolic AI approach (38%) and are based on ontologies in most articles (88%).

However, most of the AI publications related to AI in OB/GYN (82%) remain out of the scope of the usual OB/GYN journals. Additionally, formally validated AI contributions reported to date suffer from an overall poor level of validation (one data set in most cases and no external validation in all cases) and actual AI contributions remain at the level of “proof of concept” or “proof of feasibility.”

### Publications in OB/GYN Discipline Journals

The reported AI contribution to OB/GYN in the core discipline journals was 18% in comparison with 82% in journals of other disciplines. This can be explained by the early stage of research in AI or by the absence of clinical validation, meaning that the results are more relevant for the AI and computer science community. When novel algorithms are developed, computer science journals are naturally preferred [80-84]; for example, one of the first convolutional neural networks able to perform automated plane recognition during a fetal ultrasound scan was reported in a computer science journal [85]. In addition a clinically validated ML-based fetal biometric system was reported in a general medical imaging journal, not in a core OB/GYN discipline journal [86]. Another contribution based on logic and semantic reasoning for early pregnancy diagnosis was reported in a medical informatics journal [87]. These examples illustrate that core OB/GYN discipline journals await clinical value demonstration of AI-based research rather than reporting on novel systems. This pattern might also suggest that the time has come for the OB/GYN community to take over some valuable early-stage AI contributions within its core discipline journals.

Additionally, we have observed more advanced AI techniques and architectures applied to OB/GYN in computer science journals than in OB/GYN discipline journals. Moreover, the well-established and most robust ANN architectures (eg, U-net, ResNet) are no longer published in computer science journals and are largely published in OB/GYN discipline journals to present another application context [39,41,44]. As a result, a strong representation of experts in AI methods in editorial boards could improve editorial choices, which would help to fill in the delay of translation of advanced AI to the OB/GYN readership.

Interestingly, reported AI methods are applied in unconnected data silos in the field of OB/GYN (images, omics data, clinical data, other data modalities) and mixed AI methods in the field of OB/GYN are in early stages. Thus, approaches involving both ML and knowledge bases is a new direction that we expect to emerge. For example, the Smart Ultrasound in Obstetrics and Gynecology (SUOG) initiative (EIT-Health Innovation program) [88] combines knowledge bases for differential diagnosis and ML for image analysis to develop an AI-based ultrasound diagnosis assistant.

### Quality of AI Research Reporting in the OB/GYN Field

The low level of validation of AI processes in medicine has been previously reported [89]. We also observed significant heterogeneity in the description of AI processes in this review, with an overall limited level of description in most publications and with a poor level of clinical validation. This can be explained because, until recently, there were no AI-specific guidelines for medical publications. Indeed, most AI notions are new to the medical readership, medical editorial boards, and medical literature indexing. Some medical publications have proposed glossaries and definitions of basic AI notions, and the first reference guidelines for reporting medical studies involving AI were published in 2020 [90-95]. Although these initiatives should improve the reporting of AI-related publications, these guidelines only cover ML approaches. For example, the extension of SPIRIT (Standard Protocol Items: Recommendations for Interventional Trials) guidelines for clinical trial protocols using interventions involving AI (SPIRIT-AI) [92] lists the items of interest for AI publications but does not cover knowledge representations, ontologies, semantic reasoning, nor knowledge bases. In addition, we found that 38% of the articles in this review leverage these AI approaches. Consequently, a further extension of these recommendations could advantageously provide guidelines for symbolic AI approaches.

Albeit not covered in the guidelines for AI-related research, some “routine” methods in statistics (eg, logistic regression, multivariate logistic regression) and in data visualization (eg, K-means clustering) are also considered as ML approaches [96]. In this review, we excluded studies based on these methods [97-99]. However, from a perspective of consistency, some statistical methods involving ML techniques could also be covered by AI-related research guidelines.

There are recurrent debates on ethical and legal considerations in AI methods in the news and social media; therefore, we were surprised that most publications do not elaborate on these aspects. The majority use nonexplainable approaches such as ANNs; while using such nonexplainable methods is acceptable, some limitations need disclosure, and their reproducibility needs proper assessment. The most straightforward assessment method of reproducibility relies on external validation, which remains critical prior to application of all methods, but even more so if nonexplainable. Human responsibility in using AI-based processes also depends on the level of autonomy of the process [100] and on recommendations to use such processes [101].

### Limitations of MeSH Indexation in PubMed

This is the first systematic review on AI contributions reported in OB/GYN core journals. This study was performed by a pluridisciplinary group of experts from both the OB/GYN and computer science communities [102]. We have limited our paper selection to citations in PubMed and used the science disciplines as defined by WoS/JCR, thus controlling potential bias in the definition of journal domains. Although our method is reproducible and complies with systematic review guidelines, it is by essence subject to bias in publication indexation. For example, articles with ML methods mentioned only in one paragraph (eg, [103]) are not covered in this study. In addition, for papers with a scope in decision support (eg, [104,105]), the indexation will not fall under the MeSH term “artificial intelligence” in PubMed but rather under the MeSH term “diagnosis, computer-assisted” that is a distinct notion. However, unlike systematic reviews of clinical therapies, this limitation is less of a problem as we were still able to ascertain general trends in this relatively novel field of study.

All reviewed papers on robotic surgery were indexed in PubMed with the MeSH term “robotics” and under the MeSH term “artificial intelligence.” Currently, in MeSH, “robotics” is a subcategory of “artificial intelligence.” As a result, all robotic surgery papers are considered to be AI papers, which is not always the case. A revision of MeSH terms and/or indexation policies could be a solution for disambiguation. Additionally, the use of appropriate AI-oriented keywords provided by authors at the time of submission could improve the characterization of AI-based research.

### Conclusions

Until mid-2020, mostly preliminary work in AI applied in OB/GYN has been reported and published outside the usual OB/GYN journals. When published in OB/GYN journals, multiple data set validation and clinical validation of AI processes remain unmet prerequisites. Clarification in AI methods could be achieved by improving the MeSH indexing of publications in PubMed. Additionally, the reporting of AI applications should be improved by the new 2020 guidelines and recommendations in medical research involving AI. These are promising for future clinically relevant and methodologically valid clinical trials publications. However, these guidelines are covering only a part of AI approaches involved in the articles reviewed in this study, and updates need to be considered, especially to cover symbolic AI approaches.

## Appendix

Multimedia Appendix 1 PRISMA (Preferred Reporting Items for Systematic Reviews and Meta-Analyses) checklist.

Multimedia Appendix 2 List of journals covered by the review, by science discipline (source Web of Science) and grouped by discipline categories.

## Abbreviations

AI: artificial intelligence
ANN: artificial neural network
GOFAI: Good Old-Fashioned Artificial Intelligence
JCR: Journal Citation Reports
MeSH: Medical Subject Heading
ML: machine learning
OB/GYN: obstetrics and gynecology
PRISMA: Preferred Reporting Items for Systematic Reviews and Meta-Analyses
SPIRIT-AI: Standard Protocol Items: Recommendations for Interventional Trials involving Artificial Intelligence
SUOG: Smart Ultrasound in Obstetrics and Gynecology
WoS: Web of Science
