# Supplementary material for: Contributions of Artificial Intelligence Reported in Obstetrics and Gynecology Journals: Systematic Review
Source: J Med Internet Res. 2022 Apr 20;24(4):e35465. doi: 10.2196/35465 (PMC9069308; doi:10.2196/35465)
Supplement: Multimedia Appendix 2 [file jmir_v24i4e35465_app2.docx]

Table S1. List of journals covered by the review, by science discipline (source Web of Science) and grouped by discipline categories. Some journals may belong to more than one category.

| Discipline categories | Web of Science™️ disciplines | Journals | | |
| --- | --- | --- | --- | --- |
| OB/GYN CORE DISCIPLINES | OBSTETRICS & GYNECOLOGY  DEVELOPMENTAL BIOLOGY  SURGERY  ONCOLOGY  UROLOGY & NEPHROLOGY  REPRODUCTIVE BIOLOGY  ANDROLOGY | *Acta Obstet Gynecol Scand*  *Am J Obstet Gynecol*  *Am J Perinatol*  *Anticancer Res*  *Arch Esp Urol*  *Arch Gynecol Obstet*  *Asian J Androl*  *Best Pract Res Clin Obstet Gynaecol*  *Biol Reprod*  *Birth Defects Res*  *Birth Defects Res C Embryo Today*  *BJOG*  *BMC Pregnancy Childbirth*  *Breast Cancer Res*  *Clin Epigenetics*  *Clin Obstet Gynecol*  *Comput Aided Surg*  *Curr Opin Obstet Gynecol*  *Curr Opin Urol*  *Dev Cell*  *Eur J Contracept Reprod Health Care* | *Eur J Obstet Gynecol Reprod Biol*  *Female Pelvic Med Reconstr Surg*  *Fertil Steril*  *Fetal Diagn Ther*  *Ginekol Pol*  *Gynecol Endocrinol*  *Gynecol Obstet Invest*  *Gynecol Oncol*  *Hum Fertil (Camb)*  *Hum Reprod*  *Int J Comput Assist Radiol Surg*  *Int J Gynaecol Obstet*  *Int J Med Robot*  *Int J Obstet Anesth*  *Int Urogynecol J*  *J Assist Reprod Genet*  *J Endourol*  *J Gynecol Obstet Hum Reprod*  *J Matern Fetal Neonatal Med*  *J Minim Invasive Gynecol*  *J Obstet Gynaecol*  *J Obstet Gynecol Neonatal Nurs*  *J Obstet Gynaecol Res* | *J Pediatr Surg*  *J Perinat Med*  *J Reprod Med*  *J Urol*  *JSLS*  *Mol Hum Reprod*  *Mol Med Rep*  *Neurosurg Focus*  *Obstet Gynecol*  *Paediatr Perinat Epidemiol*  *Pediatr Neurosurg*  *Placenta*  *Reprod Biol*  *Reprod Biol Endocrinol*  *Reprod Toxicol*  *Reproduction*  *Surg Endosc*  *Syst Biol Reprod Med*  *Taiwan J Obstet Gynecol*  *Theriogenology*  *Ultrasound Obstet Gynecol*  *Zygote* |
| OTHER MEDICAL CLINICAL DISCIPLINES | ANESTHESIOLOGY  AUDIOLOGY & SPEECH-LANGUAGE PATHOLOGY  CARDIAC & CARDIOVASCULAR SYSTEMS  CLINICAL NEUROLOGY  ENDOCRINOLOGY & METABOLISM  MEDICINE. GENERAL & INTERNAL  NURSING  ORTHOPEDICS  OTORHINOLARYNGOLOGY  PEDIATRICS  REHABILITATION  SUBSTANCE ABUSE  TOXICOLOGY | *Alcohol*  *Am J Perinatol*  *Am J Prev Med*  *Birth Defects Res*  *Birth Defects Res C Embryo Today*  *BMC Pediatr*  *Diabetologia*  *Domest Anim Endocrinol*  *Endocrinology*  *Environ Mol Mutagen*  *Eur Cell Mater*  *Eur J Endocrinol*  *Gen Comp Endocrinol*  *Gynecol Endocrinol* | *IEEE Trans Neural Syst Rehabil Eng*  *Int J Audiol*  *Int J Obstet Anesth*  *J Chin Med Assoc*  *J Endocrinol*  *J Eval Clin Pract*  *J Korean Med Sci*  *J Neuroeng Rehabil*  *J Obstet Gynecol Neonatal Nurs*  *J Pediatr Surg*  *J Perinat Med*  *J Voice*  *JAMA Pediatr*  *Medicine (Baltimore)* | *Midwifery*  *Mol Endocrinol*  *Neurosurg Focus*  *Neurotoxicol Teratol*  *Nurs Res*  *Paediatr Perinat Epidemiol*  *Pediatr Cardiol*  *Pediatr Neurosurg*  *Pediatr Res*  *Reprod Biol Endocrinol*  *Reprod Toxicol*  *Wien Klin Wochenschr*  *Yonsei Med J* |
| MEDICAL NON-CLINICAL DISCIPLINES | CELL & TISSUE ENGINEERING  HEALTH CARE SCIENCES & SERVICES  MATHEMATICAL & COMPUTATIONAL BIOLOGY  PUBLIC. ENVIRONMENTAL & OCCUPATIONAL HEALTH  IMMUNOLOGY  MEDICINE. LEGAL  MEDICAL LABORATORY TECHNOLOGY  MEDICINE. RESEARCH & EXPERIMENTAL  MICROBIOLOGY  PATHOLOGY  PHARMACOLOGY & PHARMACY  PHYSIOLOGY | *Adv Med Sci*  *Alcohol*  *Am J Epidemiol*  *Am J Prev Med*  *Am J Public Health*  *Ann Epidemiol*  *Bioinformatics*  *Biom J*  *Biomed Pharmacother*  *Biomed Res Int*  *Biometrics*  *Biostatistics*  *Cad Saude Publica*  *Cell Physiol Biochem*  *Clin Chem Lab Med*  *Clin Chim Acta*  *CNS Neurosci Ther*  *Comput Biol Med*  *Comput Math Methods Med*  *Database (Oxford)*  *Environ Res*  *Epidemiology*  *Eur Cell Mater* | *Eur J Contracept Reprod Health Care*  *Eur J Epidemiol*  *Expert Opin Drug Deliv*  *Expert Rev Mol Med*  *Front Cell Infect Microbiol*  *Health Informatics J*  *IEEE J Biomed Health Inform*  *IEEE Trans Inf Technol Biomed*  *In Vivo*  *Int J Med InformInt J Mol Med*  *Int J Nanomedicine*  *Int J Numer Method Biomed Eng*  *J Autoimmun*  *J Biomed Semantics*  *J Cell Mol Med*  *J Cell Physiol*  *J Clin Lab Anal*  *J Eval Clin Pract*  *J Forensic Sci*  *J Healthc Eng*  *J Med Internet Res*  *J Med Syst* | *J Pharm Biomed Anal*  *J Transl Med*  *JCI Insight*  *Math Biosci*  *Med Biol Eng Comput*  *Med Decis Making*  *Med Sci Monit*  *Mol Med Rep*  *Paediatr Perinat Epidemiol*  *Pathology*  *Physiol Genomics*  *Physiol Meas*  *PLoS Comput Biol*  *Public Health Genomics*  *Qual Manag Health Care*  *Rev Saude Publica*  *Simul Healthc*  *Stat Med*  *Stat Methods Med Res*  *Stem Cell Reports*  *Technol Health Care*  *Trends Pharmacol Sci* |
| ENGINEERING DISCIPLINES | ENGINEERING. BIOMEDICAL  ENGINEERING. ELECTRICAL & ELECTRONIC | *Ann Biomed Eng*  *Artif Intell Med*  *Australas Phys Eng Sci Med*  *Biomed Eng Online*  *Biomed Mater Eng*  *Biomed Tech (Berl)*  *Comput Biol Med*  *Comput Methods Programs Biomed* | *Eur Cell Mater*  *IEEE Trans Biomed Eng*  *IEEE Trans Med Imaging IEEE Trans Neural Syst Rehabil Eng*  *IEEE Trans Pattern Anal Mach Intell*  *Int J Comput Assist Radiol Surg*  *Int J Numer Method Biomed Eng*  *J Neural Eng* | *J Neuroeng Rehabil*  *Med Biol Eng Comput*  *Med Image Anal*  *Phys Med Biol*  *Physiol Meas*  *Technol Health Care* |
| COMPUTER SCIENCE DISCIPLINES | AUTOMATION & CONTROL SYSTEMS  COMPUTER SCIENCE. ARTIFICIAL INTELLIGENCE  COMPUTER SCIENCE. CYBERNETICS  COMPUTER SCIENCE. INFORMATION SYSTEMS  COMPUTER SCIENCE. INTERDISCIPLINARY APPLICATIONS  COMPUTER SCIENCE. THEORY & METHODS | *Artif Intell Med*  *Comput Biol Chem*  *Comput Biol Med*  *Comput Methods Programs Biomed*  *IEEE J Biomed Health Inform* | *IEEE Trans Cybern*  *IEEE Trans Inf Technol Biomed*  *IEEE Trans Med Imaging*  *IEEE Trans Pattern Anal Mach Intell*  *Int J Med Inform* | *Int J Neural Syst*  *J Biomed Inform*  *Med Biol Eng Comput*  *Med Image Anal* |
| MEDICAL INFORMATICS DISCIPLINE JOURNALS | MEDICAL INFORMATICS | *Artif Intell Med*  *Biomed Tech (Berl)*  *BMC Med Inform Decis Mak*  *Comput Methods Programs Biomed*  *Health Informatics J*  *IEEE J Biomed Health Inform* | *IEEE Trans Inf Technol Biomed*  *Int J Med Inform*  *J Biomed Inform*  *J Eval Clin Pract*  *J Med Internet Res*  *J Med Syst* | *Med Biol Eng Comput*  *Med Decis Making*  *Stat Med*  *Stat Methods Med Res* |
| MEDICAL GENETICS/BIOLOGY DISCIPLINES | BIOLOGY  CELL BIOLOGY  GENETICS & HEREDITY | *Biometrics*  *Biosci Rep*  *BMC Genomics*  *BMC Med Genomics*  *Cell Mol Biol (Noisy-le-grand)*  *Cell Physiol Biochem*  *Clin Epigenetics*  *Comput Biol Chem*  *Comput Biol Med*  *Cytometry A*  *Dev Cell* | *DNA Cell Biol*  *Environ Mol Mutagen*  *Epigenetics*  *Epigenetics Chromatin*  *Epigenomics*  *Eur J Hum Genet*  *Front Biosci*  *Funct Integr Genomics*  *G3 (Bethesda)*  *Gene*  *Genes Genomics* | *J Assist Reprod Genet*  *J Cell Mol Med*  *J Cell Physiol*  *Math Biosci*  *Mol Cell Biol*  *OMICS*  *Physiol Genomics*  *Public Health Genomics*  *Stem Cell Reports*  *Zygote* |
| MEDICAL IMAGING DISCIPLINES | NEUROIMAGING  RADIOLOGY. NUCLEAR MEDICINE & MEDICAL IMAGING | *Clin Radiol*  *Diagn Interv Imaging*  *Eur Radiol*  *IEEE Trans Med Imaging*  *Int J Comput Assist Radiol Surg* | *Magn Reson Imaging*  *Med Image Anal*  *J Clin Ultrasound*  *J Ultrasound Med*  *Med Phys* | *Neuroimage*  *Phys Med Biol*  *Ultrasound Med Biol*  *Ultrasound Obstet Gynecol* |
| OTHER SCIENCE DISCIPLINES | ACOUSTICS  AGRICULTURE. DAIRY & ANIMAL SCIENCE  BIOCHEMICAL RESEARCH METHODS  BIOCHEMISTRY & MOLECULAR BIOLOGY  BIOPHYSICS  BIOTECHNOLOGY & APPLIED MICROBIOLOGY  CHEMISTRY. ANALYTICAL  CHEMISTRY. MULTIDISCIPLINARY  ENVIRONMENTAL SCIENCES  FOOD SCIENCE & TECHNOLOGY  IMAGING SCIENCE & PHOTOGRAPHIC TECHNOLOGY  MATERIALS SCIENCE. BIOMATERIALS  MATHEMATICS. INTERDISCIPLINARY APPLICATIONS  MULTIDISCIPLINARY SCIENCES  NANOSCIENCE & NANOTECHNOLOGY  NEUROSCIENCES  OPTICS  ROBOTICS  STATISTICS & PROBABILITY  VETERINARY SCIENCES  ZOOLOGY | *Acta Biochim Biophys Sin (Shanghai)*  *Animal*  *Annu Rev Anim Biosci*  *Biochem Biophys Res Commun*  *Biochim Biophys Acta Proteins Proteom*  *Bioinformatics*  *Biom J*  *Biomed Mater Eng*  *Biomed Res Int*  *Biometrics*  *Biosci Rep*  *Biostatistics*  *BMC Genomics*  *Brain Res*  *Cell Mol Biol (Noisy-le-grand)*  *Chemosphere*  *CNS Neurosci Ther*  *Cytometry A*  *DNA Cell Biol*  *Domest Anim Endocrinol*  *Environ Mol Mutagen*  *Environ Res*  *Epigenetics*  *Eur Cell Mater* | *Eur J Hum Genet*  *Eur J Neurosci*  *Expert Rev Mol Med*  *Food Funct*  *Front Biosci*  *Genes Genomics*  *IEEE Trans Med Imaging*  *Int J Mol Sci*  *Int J Nanomedicine*  *Int J Numer Method Biomed Eng*  *J Biophotonics*  *J Clin Ultrasound*  *J Dairy Sci*  *J Lab Autom*  *J Neural Eng*  *J Neuroeng Rehabil*  *J Pharm Biomed Anal*  *J Proteome Res*  *J Proteomics*  *J Ultrasound Med*  *J Vis Exp*  *Lab Chip*  *Mol Cell Biol*  *Mol Cell Proteomics*  *Nat Commun*  *Nat Neurosci* | *Neuroimage*  *Neuron*  *Neurotoxicol Teratol*  *OMICS*  *Philos Trans A Math Phys Eng Sci*  *Physiol Meas*  *PLoS Comput Biol*  *PLoS One*  *Prev Vet Med*  *Proc Natl Acad Sci U S A*  *Proteomics*  *Risk Anal*  *Sci Rep*  *Sci Total Environ*  *ScientificWorldJournal*  *Soft Robot*  *Stat Med*  *Stat Methods Med Res*  *Talanta*  *Theriogenology*  *Ultrasound Med Biol*  *Ultrasound Obstet Gynecol* |
